# Pterins as Sensors of Response to the Application of Fe^3+^-Dextran in Piglets

**DOI:** 10.3390/s100100890

**Published:** 2010-01-22

**Authors:** Miriam Smutna, Martin Svoboda, Klara Breinekova

**Affiliations:** 1 Department of Biochemistry, Chemistry and Biophysics, Faculty of Veterinary Hygiene and Ecology, University of Veterinary and Pharmaceutical Sciences Brno, Palackeho 1/3, Brno, 612 42, Czech Republic; 2 Clinic of Pig Diseases, Faculty of Veterinary Medicine, University of Veterinary and Pharmaceutical Sciences Brno, Palackeho 1/3, Brno, 612 42, Czech Republic; E-Mail: svobodama@vfu.cz; 3 Synthon s. r. o., Brnenska 32, Blansko 678 17, Czech Republic; E-Mail: Klara.Breinekova@synthon.cz

**Keywords:** iron, neopterin, biopterin, cortisol, leukogram

## Abstract

The aim of the presented study was to assess the effect of a single administration of Fe^3+^-dextran on immune cell counts and pterin biomolecule production as novel sensors of the piglets’ immune system activation, and to determine concentrations of cortisol, a traditional hormonal biosensor of the stress response. Pterins (neopterin and biopterin) in the piglets’ blood serum were analyzed by separation using reversed-phase HPLC. A single dose of Fe^3+^-dextran produced a special stress situation in the piglets’ organism which manifested itself by an increased production of neopterin (p < 0.05) and biopterin (p < 0.01) in the experimental piglets. Changes in cortisol concentrations and leukocyte counts were influenced by handling stress and were not specifically correlated to iron dextran application. Iron concentrations in the internal environment of the experimental piglets’ group were higher by an order of magnitude compared with the controls, and the highest serum concentrations of iron (p < 0.01) were reached 24 h following Fe^3+^-dextran administration. The data presented offer a new perspective on the evaluation of stress situations in the animal organism and, not least importantly, extends the rather modest current list of references on the role of pterins in livestock animals.

## 1. Introduction

Iron plays an important role in the proliferation and differentiation of immunocompetent cells. Iron ions, on the other hand, may catalyze the formation of highly toxic hydroxyl radicals in the so-called Fenton reaction. During the immune response, iron is being assimilated by cells and its bioavailable quantity is thus being reduced. This process enhances macrophage sensitivity to cytokines in general and to IFN-γ in particular. Pro-inflammatory cytokines such as TNF-α, IL-1 and IL-6 stimulate the production of ferritin, and monocyte/macrophage activation causes a decrease in the levels of iron ions in blood [1].

Biopterin is produced by nonenzymatic oxidation of tetrahydrobiopterin (BH4), and the tetrahydropterin and neopterin biosynthesis starts *de novo* by hydrolytic degradation of guanosine triphosphate (GTP) by means of guanosine triphosphate cyclohydrolase I enzyme [2].

In human medicine, research on biopterins has focused on their monitoring in, e.g., autistic children [3], or, in a different direction, in blood components obtained from the periphery blood of leukaemia or polycythaemia vera patients [4]. It follows from the results of a study by Hashimoto *et al.* [5] that biopterin concentrations may also be affected by depression and physical load (stress). The authors of the study hypothesize that most of human plasma biopterin comes from the adrenal gland, the sympathetic nerves where (BH4) is the essential cofactor of the hydroxylases of aromatic amino acids (Phe, Tyr) that participate in catecholamine synthesis. Last but not least, biopterin is the cofactor of all three forms of NO synthase (NOS). The enzyme contains iron and produces a group of nitrogenous substances conventionally called NO·s (nitric oxides). Macrophages can effectively utilize nitric oxides together with reactive oxygen metabolites to dispose of exogenous material.

In human medicine, neopterin is used more frequently as a biomarker for a number of diseases. Elevated neopterin concentrations have been reported in association with inflammatory reactions, autoimmune diseases and certain types of malignant tumours [6–8], bacterial and viral diseases, including diseases caused by protozoa, fungi and parasites [9]. Elevated neopterin concentrations have been registered even during transplant surgeries/surgical stress [10].

Because of its stability in biological samples (human serum and urine), neopterin is considered a very useful biomarker of the intensity of Th-1 type cell-mediated immune response. Murr *et al.* [8] believe that high neopterin concentrations are contingent on an increase in the production of reactive oxygen species (ROS) and, at the same time, low antioxidant concentrations (vitamin E), and they classify it as an indirect oxidation stress biomarker during the cell-mediated immune response.

Iron deficiency in piglets is a major issue. The most important factors leading to anaemia are a high intensity of growth rates in the early postnatal period, low iron levels in sows’ milk and insufficient foetal reserves of iron. Compared with other mammals, piglets are much more susceptible to iron deficiency anaemia that may even cause laboured breathing, pale skin, lethargy and rapid heart rates. Also high is the incidence of infectious diseases, particularly intestinal infections caused by *E. coli* [11].

Application of iron dextran is commonly used in piglets for the treatment or prevention of iron deficiency anaemia. Parenteral application of iron dextran is also used in human patients. Studies evaluating effects of high doses of iron dextran in humans has been so far based on *in vitro* studies only [12]. Experiments on pigs can thus provide valuable information.

## 2. Results and Discussion

### 2.1. Iron Concentrations

In the experimental group, total iron concentrations were higher by an order of magnitude compared with piglets from the control group. The highest serum iron concentrations (p < 0.01) were in the experimental group at 24 h following Fe^3+^-dextran administration (Table 1).

### 2.2. Leukocyte Counts

The average total white blood cell (WBC) counts did not differ significantly between the experimental and the control group at any time point (Table 2).

There was an increase in WBC counts in 4 hours in both groups of piglets. At 24 hours after iron dextran administration the WBC counts in the control group returned to baseline. In the experimental group the WBC counts had not yet returned to baseline values.

There was a relative shift in the composition of the leukocyte population. In both groups of piglets the percentage of lymphocytes dropped. The decrease was nevertheless greater in the experimental group. The percentage of neutrophils increased in both groups of piglets. The decrease in lymphocytes and the increase in neutrophils were greater in the experimental group. The neutrophils recovery to baseline levels was also slower in iron treated group.

Evident changes were observed in monocyte counts. After an hour, a decrease in monocyte counts inline was found in the experimental group. We found also a decrease in eosinophil counts up to 4 h in both groups of piglets. The relative shift in the leukocyte population is indicative of stress. Brohee *et al.* [13] also reported monocytopaenia, eosinopaenia, lymphopaenia and neutrophilia during acute stress. The greater increase of neutrophils in the experimental group could indicate an additional effect of iron. However this is contradictory to Brock [14], who noted a decrease in neutrophil counts at high iron concentrations.

The decrease in monocyte counts in the experimental group may have been caused by monocyte migration to the site of iron dextran administration. It follows from our results that neither leukocyte counts nor cortisol concentrations are specific sensors of the response to iron dextran application in piglets.

### 2.3. Cortisol Concentrations

Serum cortisol concentrations were determined at the first sampling (0 h) and 24 hours after Fe^3+^-dextran administration. The cortisol concentrations increased significantly in both groups.The handling stress also influenced immune cells and cortisol levels in the control group of piglets which received no iron dextran.

### 2.4. Serum Neopterin and Biopterin Concentrations

**Figure 1.**
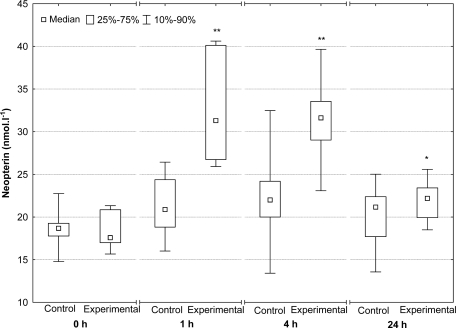
Serum neopterin concentrations in piglets which received no Fe^3+^-dextran (control group) and piglets administered Fe^3+^-dextran (experimental group; * p < 0.05, ** p < 0.01).

Mean neopterin levels in all the animals prior to the Fe^3+^-dextran administration were 18.62 ± 2.22 nmol·L^−1^. An hour after Fe^3+^-dextran administration, neopterin concentrations of experimental group piglets rose significantly (p < 0.01). Neopterin levels in the control group, on the other hand, showed no major variations during the entire experiment. In the experimental group, neopterin concentrations at the 4 hour interval were always significantly higher compared with the controls (p < 0.01). By the end of the 24 hour period, neopterin concentrations in the experimental group had dropped significantly, but they remained statistically higher compared to those of the controls (p < 0.05; Figure 1).

**Figure 2.**
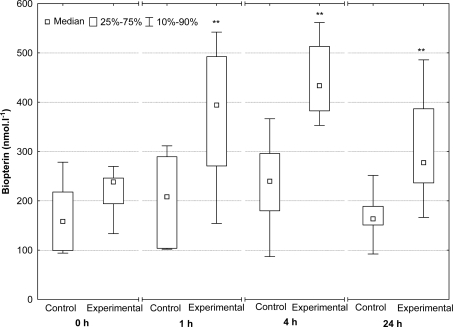
Serum biopterin concentrations in piglets which received no Fe^3+^-dextran (control group) and piglets administered Fe^3+^-dextran (experimental group; ** p < 0.01).

A similar trend was ascertained in biopterin, *i.e.*, the other of the pterins investigated. An hour after Fe^3+^-dextran administration, significantly higher serum biopterin concentrations in piglets were found compared with pre-administration levels (p < 0.01). After 4 hours, concentrations were still higher compared with the controls. At 24 hours, biopterin concentrations in the serum of experimental piglets dropped but still remained to be higher than in the control group (p < 0.01) (Figure 2).

It follows from our results that the Fe^3+^-dextran administration was followed by a short-term activation of the immune system. There is an ambivalent relationship between iron and immune functions and infection, because, on the one hand, it is an element indispensable for the performance of a number of cellular and immune functions, but, on the other, its deficit has an inhibitory effect [15]. It has been reported that an iron overdose affects immune system. In the study of Walker and Walker [16] high iron concentrations produced by an iron overdose significantly affected the immune system, where iron reduced the phagocytic capacity, affected T-lymphocyte activity and modified lymphocyte distribution at various locations of the immune system. Similarly Cardier *et al.* [17] mentioned the effects of high iron concentrations on T-lymphocyte distribution in blood, spleen and the mesenterial lymph nodes.

Iron deficiency, on the other hand, is associated with a decrease in neutrophil activity, or, rather, decreased activity of Fe^3+^-dependent enzymes, such as myeloperoxidase [18]. It also suppresses T-lymphocyte blastogenesis and mitogenesis during the immune response to various pathogens [19].

An increase in neopterin and biopterin production was observed already an hour after Fe^3+^-dextran administration. Neopterin and biopterin are closely related to the activation of the Th-1 immune system. These pterin derivatives are produced in the organism by monocytes/macrophages following stimulation by the interferon-gamma (INF-γ) cytokine, which is released by T-lymphocytes and NK cells. Biopterin synthesis also takes place in T-cells, B-cells, in the endothelium, smooth muscle cells, fibroblasts, *etc.* [20]. Neopterin functions have been the subject of many *in vitro* studies which suggest that the oxidation status may be affected by the neopterin’s pro-oxidative action [21–23]. NO synthase participating in the production of NO· requires a reduced form of biopterin for its activity. Tetrahydrobiopterin is also the cofactor for aromatic amino acid hydroxylases (PAH-phenylalanine-4-hydroxylase, tyrosine-3-hydroxylase and tryptophan-5-hydroxylase).

It is evident from our experiment that the administration of Fe^3+^-dextran may trigger acute activation of the immune system and production of pterin derivatives which are indicators of the production of pro-inflammatory cytokines (IFN-γ), NO, *etc.* Further studies with the employment of pro-inflammatory cytokines measurements will be needed to fully characterise these effects.

The variation of neopterin and biopterin values was high in both experimental and control groups. Similar variation was found also in duplicate samples. There is no comparative literature data so far presenting neopterin and biopterin levels in weaned piglets. In our opinion the variation may be explained by the individual stress responses.

There are only a few authors in veterinary medicine who studied the use of pterins in disease diagnosis. For instance, Schrodl *et al.* [24] investigated the effects of a bacterial infection (*Haemophilus parasuis*) on porcine serum neopterin levels. In their study they found a decrease in neopterin concentrations compared with the control group. In their experimental model with pigs, Amann *et al.* [25] evaluated the possibility of using neopterin to monitor heart attacks. Their results, however, suggest that neopterin is not a suitable biomarker of cardiovascular diseases in pigs. Biopterin production in the acute phase of hypoxia-ischemia in newborn piglets was monitored by Fujioka *et al.* [26] who found that while cerebral production of biopterin in newborn piglets showed no response to hypoxia-ischemia, biopterin blood plasma concentrations responded by a fivefold increase. Ziegler *et al.* [4] monitored erythrocyte biopterin concentrations in beagles in connection with bone marrow transplantations after its destruction by irradiation. Biopterin was perceived as a biomarker of haemolytic cell proliferation activity.

It follows from the above findings in the area of veterinary medicine that we have very few literary data that can be used for comparative purposes. Our study presented here has its originality, and brings a new angle to the assessment of stress situations in an animal’s organism. Last but not least, it broadens modest list of studies investigating pterins in their untraditional role of sensors of immune system activation which occurs as a response of organism’s internal environment to iron stress.

## 3. Instruments and Methods

### 3.1. Experimental Design

A total of 22 large white breed (LWB) piglets x Landrace piglets weighing 12 ± 1.15 kg on average were included in the study. The piglets were divided into two groups. Group 1 was the control group (n = 10). Group 2 was the experimental group (n = 12) where piglets received a single i.m. dose of 2.000 mg Fe^3+^-dextran (20 mL Fe^3+^-dextran per piglet, 1 mL contained 100 mg Fe^3+^, *i.e.*, 2.000 mg Fe^3+^). Blood samples were collected from piglets prior to Fe^3+^-dextran administration (0 h), and 1 hour (1 h), 4 hours (4 h) and, lastly, 24 hours (24 h) after Fe^3+^-dextran administration to the experimental group piglets. At the above intervals, blood samples were withdrawn from piglets in both the control and the experimental groups. After precipitation, the blood samples collected were centrifuged at 3.000 rpm for 15 min, and serum was frozen at −18 °C until the final analysis. Blood (serum) samples were handled out of direct sunlight and, whenever possible, in a shaded environment.

### 3.2. Haematological Examination

EDTA (ethylenediaminetetraacetic acid) was used as an anticoagulant for the haematological examination. The white blood cell count (WBC) was determined by Celtac Alpha haematology analyzer (Nihon Kohden). Differential leukocyte counts were determined after staining blood smears with May-Grünwald and Giemsa-Romanowsky solutions.

### 3.3. Determination of Iron Concentration

Heparin was used as an anticoagulant for the determination of iron concentrations in blood plasma. Blood plasma iron concentrations (Fe) were determined photometrically by measuring iron complexes with ferrozine (set Iron Bio-La-TEST^®^ PLIVA-Lachema Diagnostics, Czech Republic).

### 3.4. Sample Treatment before Pterine Determination

Prior to the actual analysis, the serum samples were properly thawed at room temperature and carefully mixed by gentle turning. For protein removal, 100 μL of 20% trichloroacetic acid were added to 500 μL of serum sample and vortexed for 10 s. After 10 min in dark at room temperature, the samples were centrifuged at 3.000 rpm for 15 min.

### 3.5. HPLC Analysis

The supernatant was filtered through a 0.45 μm nylon membrane filter, and 50 μL of the sample were injected into the HPLC system [27]. The detection was accomplished by means of Alliance 2695 chromatographic system with a fluorescent detector (Waters 2475, USA). The detection was performed at the wavelengths selected as γ_ex_ = 353 nm and γ_em_ = 438 nm. The column used was Zorbax Eclipse XDB-C18 (150 × 4.6 mm, 5-Micron; Agilent, USA). The analysis was done in an isocratic mode, using water/acetonitrile (97:3, v/v) as the mobile phase. The mobile phase flow rate was 0.9 mL·min^−1^, column temperature 35 °C. All materials were HPLC-grade purity.

### 3.6. Sample Treatment before Cortisol Determination

SPEC C_18_ AR cartridges of 3 mL with 15 mg solid-phase mass per column (Varian, USA) were used. 500 μL of the sample containing the internal standard (19-nortestosterone, final concentration 2 ng/μL) was allowed to pass through a preconditioned cartridge (500 μL methanol and 500 μL water). The cartridge was then washed with 500 μL acetone/water (10:90, v/v) and after drying the cartridge for 5 min the analyte was eluted with 1 mL acetonitrile.

### 3.7. HPLC Analysis of Cortisol

The sample volume injected into the HPLC system was 20 μL. Cortisol was separated by an isocratic elution method (65:35 acetonitrile/water, v/v) on the Zorbax Eclipse XDB-C18 column (150 × 4.6 mm, 5-Micron; Agilent, USA). The mobile phase flow rate was 1 mL·min^−1^, column temperature was 30 °C, and UV detection was done at 245 nm.

### 3.8. Validation Prameters

Peak identification was based on the comparison of the retention time with the standards for the studied biomolecules. The linearity for neopterin was measured in the concentration range of 0.6–250 ng/mL (y = 9369.5x + 2111.4; R^2^ = 0.999), for biopterin in the range of 1.6–52.0 ng/mL (y = 18602x − 7446; R^2^ = 0.999) and for cortisol in the range of 0.01–20.0 μg/mL (y = 23.71x + 538.87; R^2^ = 0.999). Neopterin detection limit was 0.5 ng/mL (3 S/N), for biopterin was 1.3 ng/mL, and for cortisol was 20.1 ng/mL (3 S/N). Determination limit for neopterin was 1.49 ng/mL (10 S/N), for biopterin 4.33 ng/mL, and for cortisol 64.6 ng/mL (10 S/N). The method’s yield was 91%, 94% and 89% for neopterin, biopterin and cortisol, respectively.

### 3.9. Statistical Evaluation

Statistical evaluation of results was carried out using Statistica software 8.0 for Windows (StatSoft). Data were first tested for normality (Kolmogorov-Smirnov test) and homoskedasticity of variance (Bartlett’s test). If those conditions were satisfied, one-way analysis of variance (ANOVA) was employed to determine whether there were any significant differences in measured variables between groups. When a difference was detected (p < 0.05), Tukey’s multiple comparison test was applied to identify which treatments were significantly different.

## 4. Conclusions

Neopterin and biopterin belong to a group of unconjugated pterin derivatives. These biomolecules are present in many animal species and perform a number of functions. Neopterin is produced primarily by immune system cells, *i.e.*, monocytes/macrophages, and, in human medicine, it is used as a reliable marker of Th-1 type of cell immunity activation. The reduced form of biopterin, *i.e.*, tetrahydrobiopterin, is an important cofactor of NO synthesis and hydroxylases of aromatic amino acids (phenylalanine hydroxylase, tryptophan hydroxylase and tyrosine hydroxylase), which play a role in catecholamine and serotonin metabolism. In veterinary medicine, however, the issue of neopterin and biopterin metabolism and their functions have neither been thoroughly studied nor understood.

In our study, increased neopterin and biopterin concentrations in serum of piglets 4 hrs after intramuscular administration of Fe^3+^-dextran compared with the control group (p < 0.01) were detected. Twenty-four hours after Fe^3+^-dextran administration, a statistically significant decrease in both biopterin and neopterin concentrations was recorded (p < 0.01 and p < 0.05, respectively).

It follows from our results that the synthesis of pterin derivatives, *i.e.*, neopterin and biopterin, is influenced by diverse stress factors. Neopterin and biopterin serum concentrations may thus provide additional information on the influence of stress factors on the porcine organism *in vivo*, and, at the same time, they extend the modest list of papers on pterins in veterinary practice.

## Figures and Tables

**Table 1.** Iron concentrations (mean ± S.D.) in serum of piglets (** p < 0.01), S.D.-standard deviation.

	**Fe (ng·mL^−1^)**			

	**0 h**	**1 h**	**4 h**	**24 h**
**Control**	26.02 ± 7.21	25.38 ± 9.10	28.15 ± 8.40	22.92 ± 6.31
**Fe^3+^-dextran group**	29.93 ± 6.26	144.30 ± 44.2 **	324.95 ± 59.80 **	331.03 ± 44.82 **

**Table 2.** Differential leukocyte counts. N/L ratio = Neutrophils /Lymphocytes.

		**0 h**	**1 h**	**4 h**	**24 h**	***p***
**Leukocytes (10^9^/L)**	Control	18.6 ± 2.9	21.1 ± 4.9	21.2 ± 4.0	18.8 ± 4.4	0.05
	Fe^3+^-dextran	17.9 ± 2.9	20.9 ± 5.5	21.3 ± 7.6	22.8 ± 8.9	0.05

**Neutrophils (%)**	Control	38.9 ± 19.9	59.8 ± 14.4	56.2 ± 16.8	46.8 ± 9.9	0.05
	Fe^3+^-dextran	42.1 ± 11.8	60.7 ± 9.0	64.0 ± 12.1	56.7 ± 13.0	0.05

**Lymphocytes (%)**	Control	55.2 ± 13.6	35.8 ± 13.2	41.6 ± 16.8	46.7 ± 11.1	0.05
	Fe^3+^-dextran	52.4 ± 13.2	35.7 ± 9.1	33.3 ± 14.6	38.9 ± 15.0	0.05

**Monocytes (%)**	Control	4.5 ± 3.3	4.3 ± 3.8	1.9 ± 2.2	4.8 ± 5.1	0.05
	Fe^3+^-dextran	4.5 ± 2.4	2.8 ± 1.8	2.4 ± 2.6	3.7 ± 2.7	0.05

**Eosinophils (%)**	Control	1.1 ± 0.8	0.1 ± 0.3	0.3 ± 0.6	1.8 ± 1.7	0.05
	Fe^3+^-dextran	0.9 ± 0.9	0.6 ± 1.0	0.2 ± 0.6	0.5 ± 1.0	0.05

**N/L ratio**	Control	0.70	1.67	1.35	1.00	0.05
	Fe^3+^-dextran	0.80	1.70	1.92	1.46	0.05

p = N.S.

**Table 3.** Piglet serum cortisol concentrations (mean ± S.D.) prior to (0 h) and 24 hours after the administration of a single dose of Fe^3+^-dextran. (** p < 0.01), S.D.-standard deviation.

**Sampling**	**Cortisol (nmol·L^−1^)**	
	**Control group**	**Fe^3+^-dextran group**
**0 h**	164. 29 ± 62.00	218.14 ± 80.59
**24 h**	502.39 ± 89.35 **	501.56 ± 157.77 **
